# Growth and Element Uptake by Salt-Sensitive Crops under Combined NaCl and Cd Stresses

**DOI:** 10.3390/plants10061202

**Published:** 2021-06-12

**Authors:** Gabrijel Ondrasek, Zed Rengel, Nada Maurović, Nada Kondres, Vilim Filipović, Radovan Savić, Boško Blagojević, Vjekoslav Tanaskovik, Cristian Meriño Gergichevich, Davor Romić

**Affiliations:** 1 Department of Soil Amelioration, Faculty of Agriculture, University of Zagreb, 10000 Zagreb, Croatia; nmaurovic@agr.hr (N.M.); nkondres@agr.hr (N.K.); vfilipovic@agr.hr (V.F.); dromic@agr.hr (D.R.); 2 School of Earth and Environment, University of Western Australia, Perth 6009, Australia; zed.rengel@uwa.edu.au; 3 Institute for Adriatic Crops and Karst Reclamation, 21000 Split, Croatia; 4 Department of Water Management, Faculty of Agriculture, University of Novi Sad, 21102 Novi Sad, Serbia; radovan.savic@polj.uns.ac.rs (R.S.); bosko.blagojevic@polj.uns.ac.rs (B.B.); 5 Faculty of Agricultural Sciences and Food, University of Ss. Cyril and Methodius in Skopje, 1000 Skopje, North Macedonia; vjekoslavtanaskovic@yahoo.com; 6 Departamento de Producción Agropecuaria, Facultad de Ciencias Agropecuarias y Forestales, Universidad de La Frontera, Temuco 1145, Chile; cristian.merino@ufrontera.cl

**Keywords:** NaCl stress, metal stress, salinity, soil Cd contamination, glycophytes, K/Na, Ca/Na, Mg/Na, strawberry, lettuce

## Abstract

To test an assumption that organic soil can ameliorate nutritional disorders associated with metal and salinity stresses, we exposed salt-sensitive strawberry and lettuce to four salinity (0–60 mM NaCl) and three contamination (0.3–5 mg Cd/kg) rates in peat (pH_H2O_ = 5.5). The results showed that, even at 20 mM NaCl, salinity stress exerted a dominant effect on rhizosphere biogeochemistry and physiological processes, inducing leaf-edge burns, chlorosis/necrosis, reducing vegetative growth in crops; at ≥40 mM, NaCl mortality was induced in strawberry. Signifiacntly decreased K/Na, Ca/Na and Mg/Na concentration ratios with raising salinity were confirmed in all tissues. The combined CdxNaCl stresses (vs. control) increased leaf Cd accumulation (up to 42-fold in lettuce and 23-fold in strawberry), whereas NaCl salinity increased the accumulation of Zn (>1.5-fold) and Cu (up to 1.2-fold) in leaves. Lettuce accumulated the toxic Cd concentration (up to 12.6 mg/kg) in leaves, suggesting the strong root-to-shoot transport of Cd. In strawberry Cd, concentration was similar (and sub-toxic) in fruits and leaves, 2.28 and 1.86 mg/kg, respectively, suggesting lower Cd root-to-shoot translocation, and similar Cd mobility in the xylem and phloem. Additionally, the accumulation of Cd in strawberry fruits was exacerbated at high NaCl exposure (60 mM) compared with lower NaCl concentrations. Thus, in salinized, slightly acidic and organically rich rhizosphere, pronounced organo- and/or chloro-complexation likely shifted metal biogeochemistry toward increased mobility and phytoavailability (with metal adsorption restricted due to Na^+^ oversaturation of the caton exchange complex in the substrate), confirming the importance of quality water and soils in avoiding abiotic stresses and producing non-contaminated food.

## 1. Introduction

Increased water scarcity, accompanied by climate change, necessitates the use of marginal (*grey*) hydro-resources, often loaded with various pollutants [1], especially salts and heavy metals [2,3]. Consequently, important environmental resources, notably quality lands and waters, have become increasingly limiting for food production over recent decades due to excessive salinization [4] and/or metal contamination [5,6]. Globally, ~20% of irrigated agroecosystems provide ~33% of food supply, but are frequently salt-affected, mostly with excessive Na and Cl ions [3,7]. Horticultural glycophytes are particularly vulnerable to excessive salinity, whereby salt stress limits plant growth and reduces crop yield and quality [2,8,9]. Depending on stress severity and duration, as well as crop, genotype and developmental stage, salt stress disturbs (i) water relations, i.e., osmotic potential [2,10], (ii) ion/mineral homeostasis [11] and (iii) a wide range of metabolic (e.g., hormonal, enzymatic, antioxidant) processes, causing secondary stresses [7].

Soil metal contamination represents a serious and long-term threat to ecosystem services. Most food crops are sensitive to increased concentration of metals that can easily be taken up and deposited in edible tissues. The consumption of food, cultivated on metal-contaminated areas, or irrigated with metal-enriched water resources, represents one of the most dominant transmission routes for many potentially toxic metals into the human population [6,12]. Among metals, special attention is paid to Cd, due to its toxicity and rapid root-to-shoot translocation. The most dominant parameters affecting Cd biogeochemistry and its soil-plant transfer are soil pH [6], concentration of inorganic ligands (especially Cl ions) [12], organic matter (OM) content [13], and their interactions. For instance, we recently reported on the significant (*p* < 0.01) NaClxCd interaction enhancing Cd transfer to edible radish tissue grown in OM-depleted mineral soil, containing increased concentration of Cd–Cl complexes in the rhizosphere [14]. In contrast, [13] recorded the significant (*p* < 0.01) NaClxCd interaction on CaCl_2_-extractable Cd in the OM-amended rhizosphere, but with reduced Cd phytoavailability and uptake by faba bean, accompanied by an increased proportion of Cd–OM complexes in the rhizosphere.

Due to the high content of OM (>90%) and huge adsorptive interfaces (cation exchange capacity >90 cmol(+)/kg), organic soils (peats) have recently been confirmed as very effective sorbents for the immobilisation or removal of Cd (as well as Zn, Pb, Ni, Cr and As) from contaminated soils [5,15] or aqueous ecosystems [16,17], thus decreasing metal availability and reducing phyto-accumulation [5]. Consequently, the NaCl and Cd interactions are likely to differ in OM-rich soils, compared with mineral OM-depleted soils, differentially influencing metal biogeochemistry and crop uptake.

To test this hypothesis, we conducted two controlled pot trials with strawberry and lettuce, two of the widely consumed and cultivated horticultural crops in irrigated agroecosystems, both very sensitive to salinity [18,19] and without the capacity to accumulate metals (Cd). The aim of the study was to elucidate the impact of naturally relevant abiotic stresses (combining four NaCl (0, 20, 40 and 60 mM) and three Cd (0.3, 2.5 and 5.0 mg/kg) levels) on the growth and mineral composition of test crops.

## 2. Results and Discussion

### 2.1. Vegetative Growth and Fresh Yield Parameters under Applied Treatments

**Figure 1:**
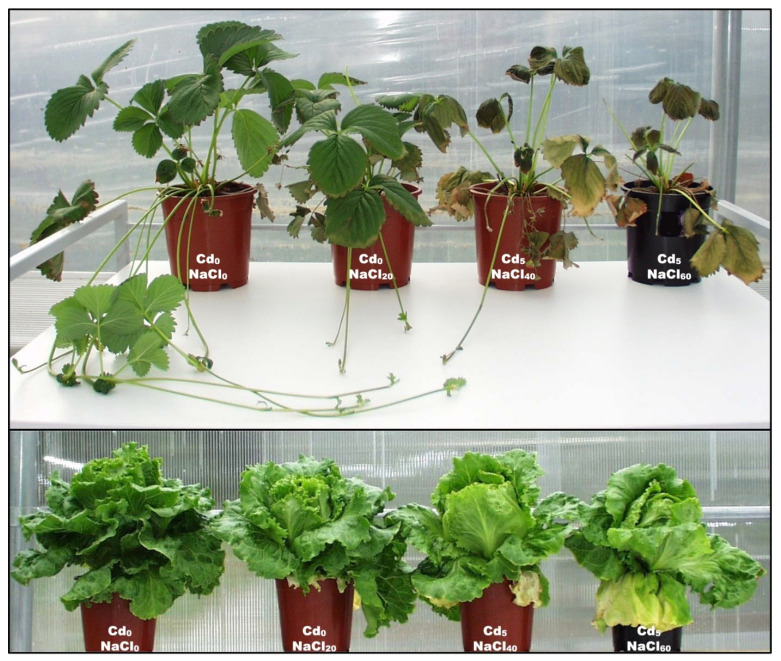
Impact of selected treatments (from left to right: Control—Cd_0_xNaCl_0_; Cd_0_xNaCl_20_; Cd_5_xNaCl_40_; Cd_5_xNaCl_60_) on growth of strawberry (70 days after salinity treatment commenced (DASAL)) and lettuce (30 DASAL).

The summary of two-way ANOVA results for vegetative growth and yield parameters is presented in Table S1. The CdxNaCl interaction was not significant for any of the vegetative growth and yield parameters measured 30 days (in case of lettuce) and 70 days after salinity (DASAL) treatment commenced (in the case of strawberry; Table S1). The NaCl main effect was significant for all the measured parameters, but the Cd effect significantly influenced only the number and length of strawberry runners and, importantly, the marketable yield (strawberry fruit biomass and lettuce head biomass) (Table 1; Figure 1). For instance, in comparison with the control (NaCl_0_), in the salt treatments, total marketable strawberry yield was reduced by up to 59%, total number of marketable fruits by up to 43%, leaf area by more than 2.1-fold, number of runners by up to 7-fold, and the length of the longest runner by up to 62% (Table 1). In addition, accelerated leaf senescence and plant mortality were noted at ≥40 mM NaCl (Figure 1). In lettuce, marketable yield was reduced by 10% (in Cd_5_) and 26% (in NaCl_60_) compared with the non-contaminated (Cd_0_) and non-salinized (NaCl_0_) controls, respectively (Table 1), and was accompanied by leaf chlorosis and leaf-edge necrosis, but without plant mortality (Figure 1). These findings are in agreement with some reports on other glycophytes, such as rice [20], muskmelon [21], pigeonpea [22] and strawberry [18], indicating that cell division and elongation were suppressed in the test crops [10], and the other vital functions were compromised, likely due to oxidative stress (discussed below).

Crop yield is the most important agricultural criterion for tolerance to different abiotic stresses, including salt [23] and metal (Cd) stresses (discussed below). In glycophytes, the relationship between salinity and yield can be expressed as a negative linear response equation at salinities above the critical threshold limit, which in our study started even at the lowest level of 20 mM NaCl (significant in strawberry) (Table 1; Figure 1). Differences between species and growth duration to achieve a marketable product resulted in different treatment effects (Table 1), including visible symptoms as withering, necrotic spots, leaf edge burn, accelerated leaf senescence (Figure 1), and vegetative and reproductive growth of strawberry being shorter by 14 (in NaCl_20_ treatment) and 22 days (in NaCl_40_ and NaCl_60_ treatments) in comparison to the non-stressed control (data not shown).

An indicative deleterious effect of elevated salt stress in glycophytes, such as strawberry, is leaf senescence, due to a decrease in chlorophyll content and perturbation of membrane permeability/selectivity [24] and, in extreme cases, plant mortality would occur. We did not notice these effects in lettuce, probably due to shorter vegetation/salt stress exposure to achieve marketable product (30 days for lettuce vs. 70 days for strawberry); however, the involvement of some other potential salt tolerance mechanisms in lettuce cannot be excluded. Forty-two days after the imposition of salinity stress to strawberry in the treatment with most severe combined stresses (Cd_5_xNaCl_60_), we observed the chlorosis of basal (oldest) leaves which, over time, gradually progressed to necrosis (Figure 1). Similar observations in field conditions were made by [18], who noticed different survival rate among five strawberry varieties at 2.5 dS/m soil salinity, from the highest of 94% in the most salt-tolerant cv. to only 53% in the most salt-sensitive cv., accompanied by chlorosis/necrosis on the oldest leaves, as a result of Cl accumulation in leaf hydathodes (pores involved in guttation). Recently, [10] reported that NaCl stress caused damage to the epidermis and the xylem of sorghum seedlings. Salt stress can induce cell death by depleting cytosolic K^+^ beyond a threshold level; thus, maintaining optimal ratios of Na^+^ to K^+^ (as well as to Ca^2+^ and Mg^2+^) in the cytosol is critical not only for the normal functioning of the cytoplasm [25], but also for avoiding (i) ion-specific deleterious impacts associated with excessive Na [26], Cl and Cd accumulation, as well as (ii) numerous secondary disorders caused by oxidative stress.

Oxidative stress is a secondary effect of salt and/or other stresses, such as drought [8] or metal toxicity (e.g., Cd, discussed in Section 2.4). Oxidative stress is caused by imbalance in the generation and decomposition of reactive oxygen species—ROS (hydrogen peroxides, hydroxyl radical, superoxide anion)—responsible for membrane damage [27,28]. In response to oxidative stress, the antioxidant enzyme activities increase to enhance the decomposition of accumulated ROS [2,22,29,30]. Especially in strawberry, as one of the most salt-sensitive fruit crops, the accumulation of ROS is an important factor that hastens fruit senescence (see Figure 1), given the ability of ROS to cause lipid peroxidation, destroy the structure and function of membranes, and cause electron leakage [31].

### 2.2. Dry Matter Content and Na and Cl Uptake as Influenced by the Applied Treatments

The ANOVA summary for dry matter content and mineral concentration in examined test crop tissues confirmed the significance of each main effect, and the CdxNaCl interaction was significant in only one case (Na concentration in strawberry leaves at 40 DASAL) (Table S2 and Table 2). The Na concentration in strawberry leaves at 40 DASAL, was decreased due to increasing Cd contamination, but increased with an increase in the applied NaCl concentration, whereas it was unaffected by the interaction and the main Cd effect in strawberry fruits and lettuce leaves at 20 and 30 DASAL (Table 2).

As expected, rising soil NaCl salinity significantly enhanced Na and Cl accumulation in strawberry leaves (Na by more than two orders of magnitude, and Cl by 13.4 fold), fruits (Na by 36-fold and Cl by 6.6-fold), as well in lettuce leaves, especially at 30 DASAL (Na by 28-fold and Cl by 16-fold) in comparison to the control with no NaCl (Table 2). Soil contamination with Cd reduced the concentration of both salt ions (Na and Cl) in strawberry leaves at 40 DASAL, and only that of Cl in fruits, whereas, in other tissues, Cd had no effect on Na and Cl concentrations (Table 2). In addition, the main effects oppositely impacted the accumulation of micronutrients Cu and Zn, whereby (i) NaCl increased their concentration in strawberry leaves at 40 DASAL (Zn by 48%), fruits (Cu by 90%, Zn by 69%), as well as in lettuce leaves at 20 DASAL (Cu by 88%, Zn by 79%) and 30 DASAL (Cu by 120%, Zn by 152%), and (ii) Cd suppressed concentrations of Cu (in strawberry 11–17% and lettuce 10–20%) and Zn (in strawberry 15–16% and lettuce 8–18%) (Table 2).

Chloride is an essential phytonutrient [32], unlike Na, which is a beneficial element to some plant species and essential to selected C4 species [33]; however, both ions in excessive concentrations can induce numerous adverse effects (e.g., Figure 1). In general, Cl concentration in the shoots of glycophytes varies between 1 and 20 g/kg DW [32,34], but the Cl threshold toxicity level is about 4–7 and 15–50 g/kg DW for Cl-sensitive and Cl-tolerant plants, respectively [35]. In the present study, Cl concentrations were well above toxicity threshold even for Cl-tolerant plants (Table 2). In addition, excessive Na accumulation in the cytosol under salt stress is toxic to cells, impairing the uptake of K, Ca and Mg [18]. In rice, as one of the most salt-sensitive species, after 3-week exposure to salt stress, [20] recorded reduced chlorophyll content, accompanied by visible leaf injuries at treatment concentrations >50 mM Na, whereas leaf damage was not apparent at Na < 20 mM. Additionally, the same authors noticed significant positive correlations between Na and the leaf injury scores in rice varieties, suggesting that Na build-up to a toxic level was the main cause of leaf senescence, particularly of older leaves; we noted a similar observation in strawberry (Figure 1) and muskmelon [21]. Hence, both crops in the study presented here were exposed to combined toxicity of Na and Cl (some treatments also to Cd toxicity) that compromised vegetative growth and fruit yield (Figure 1; Table 1).

Plant capacity to withstand Cl toxicity is mostly related to the successful suppression of the Cl root-shoot translocation [35]. There are large differences in Cl accumulation in shoots and fruits, given that plants tend to accumulate less Cl in tissues dominantly supplied via phloem (fruits) than in leafy tissues supplied via xylem [36]. Analogously, in our study, in the treatment with the highest level of salinity, we recorded almost a 2-fold lower Cl concentration in strawberry fruits (21 g/kg) in comparison to leaves (39 g/kg) (Table 2). However, large differences exist among various cultivars/genotypes of strawberry, regarding Na and Cl uptake and redistribution under salt stress. For instance, Na significantly increased in roots (up to >140%) and petioles (up to almost three-fold), but remained relatively stable in leaves, contrary to Cl, which significantly increased in all tissues of five strawberry cultivars exposed to salinity of up to 2.5 dS/m [18]. Similarly, under salinity ranging from 1.1 to 4.4 dS/m, strawberry shoot Na increased by 1.6-fold and 3.3-fold in cultivars Albion and Chandler, respectively [37]. In general, progressively increased Na uptake under increasing salinity is followed by increasingly impaired uptake of K, Ca, Mg and some micronutrients [18].

### 2.3. Altered K/Na, Ca/Na and Mg/Na Concentration Ratios Are Strong Indicators of Na-Stress Exposure

**Figure 2:**
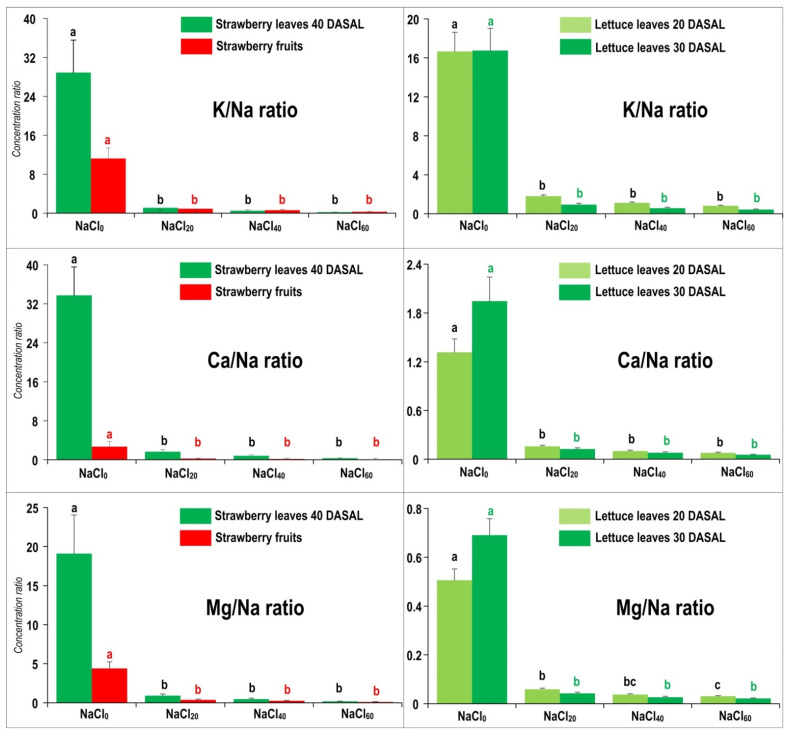
Impact of applied treatments on elemental ratios in lettuce leaves 20 and 30 days after salinity (DASAL) treatment commenced, and strawberry leaves (40 DASAL) and fruits. Bars (means + SE, *n* = 4) with the same letter and colour in each graph are not significantly different among the treatments, according to Tukey’s HSD test.

The ANOVA summary for concentration ratios of K, Ca or Mg with Na in examined test crop tissues showed a highly significant (*p* < 0.0001) impact of NaCl salinity, whereas the soil Cd contamination as well as the CdxNaCl interaction were not significant (Table S3). A sharp decrease in the ratio of a macroelement (K, Ca or Mg), and Na concentrations under applied NaCl treatments compared with the unstressed control, was recorded in all examined tissues in both crops (Figure 2). Progressive K/Na, Ca/Na and Mg/Na decreases started even at the lowest salinity level (20 mM NaCl) and followed in the order strawberry leaves > fruits > lettuce leaves, with increasing NaCl treatment concentrations (Figure 2).

Excessive NaCl salinity either in glycophyte or halophyte tissues increases the concentration of Na and Cl at the expense of K [11,20,25], Ca, Mg [10,11,38,39] and some other nutrients (NO_3_^−^, Cu and Zn). Namely, high Na and Cl concentrations in the rhizosphere soil can induce extremely low ratios of K/Na, Ca/Na, Mg/Na, NO_3_/Cl [38], as we confirmed in strawberry and lettuce (Figure 2).

Potassium (K) is the most abundant cation in plants (~100 g/kg d.w.), and strawberries are recognised as being among the fruits that are the most rich in K (~170 mg K/100 g fresh w.) [40,41]. Due to similar physicochemical properties between Na^+^ and K^+^, ion competition would start at the soil–root interface, continue onto the membrane receptors/transporters/channels, as well as regarding various cytosolic constituents and physiological processes [11,42,43]. Significantly decreased K concentration was associated with increased tissue concentrations of Na and Cl, i.e., K/Na ratio (Figure 2) decreased as a common indicator of salt stress [44]. In some plant tissues, such as roots, a low K/Na ratio appears to be a more reliable salt stress indicator than high Na concentration alone [45]. Therefore, maintaining optimal, i.e., relatively high cytosolic K^+^ concentration (~100 mM) in normal (non-stressed) conditions, and high K/Na (or K/Cl) ratio in tissues under stress conditions, is a precondition for salt-stress tolerance, ensuring the crucial functions (including cytosolic pH/volume regulation, thylakoid swelling/stacking, electron transport, photosynthetic efficiency [44,46], stomatal conductance/regulation, photorespiration, and chloroplast activity) continue with relatively little disturbance. Alternatively, the consequences would be poor photosynthetic performance and reduced growth and yield [25,41], as confirmed in the present (Figure 1) and other studies. For instance, K/Na ratio in non-stressed muskmelon plants was higher by 1–2 orders of magnitude (in leaves, peel and fruit pulp) in comparison with 60 mM NaCl-stressed plants [21]. Recently, [7] reported inhibited biomass production simultaneously with a strong decrease in K/Na ratio in cultivated and wild barley species exposed to NaCl salinity. [39] noticed significantly decreased Ca/Na ratio with increasing NaCl concentration in lucerne tissues. Similarly, [47] found that salt-tolerant lucerne cultivars absorbed more K and Ca under NaCl stress and minimized Na uptake, resulting in increased K/Na and Ca/Na ratios (contrary to salt-sensitive cultivars); however, Mg accumulation in shoots exhibited an opposite effect in comparison with Ca.

Calcium (Ca^2+^) has a crucial role in regulating Na^+^ uptake; it alleviates ionic stress by binding to the plasma membrane and blocking the non-selective cation channels that are the major pathways for Na^+^ influx in plants [10]. Additionally, exogenous Ca^2+^ ameliorates negative effects of salinity on chlorophyll and dry mass production in strawberry [48] and improves ion homeostasis and modulates the antioxidant system under salt [10,45] or metal (Al) stress [49]. The fact that shoot K/Na, Ca/Na and Mg/Na ratios were strongly decreased in both crops as NaCl salinity increased (Figure 2) may suggest that the test crops do not have membrane-bound transport proteins (e.g., high-affinity K transporters) that favour K/Ca/Mg absorption over that of Na, as was confirmed in spinach cultivars (spinach is considered more salt tolerant than strawberry and lettuce) [11]. Additionally, Ca amendment stabilized the lipid bilayer and maintained the structural integrity of cellular membranes in sorghum and rice under NaCl stress, i.e., Ca alleviated NaCl-induced oxidative damage by decreasing H_2_O_2_ content, Na/K ratio and the transcript levels of *SbAPX2*, *SbCAT3* and *SbSOS1* genes [10,50]. Recently, [51] emphasized the importance of Ca (and P) tissue concentrations in avoiding the deleterious Ca-salt precipitation reactions that would decrease the availability of macro- and micronutrients and cause physical cellular damage and cell death/leaf necrosis that occurred in our test crops exposed to the combined stresses (Figure 1).

### 2.4. Enhanced Cd Accumulation under Combined NaCl and Cd Exposure

**Figure 3:**
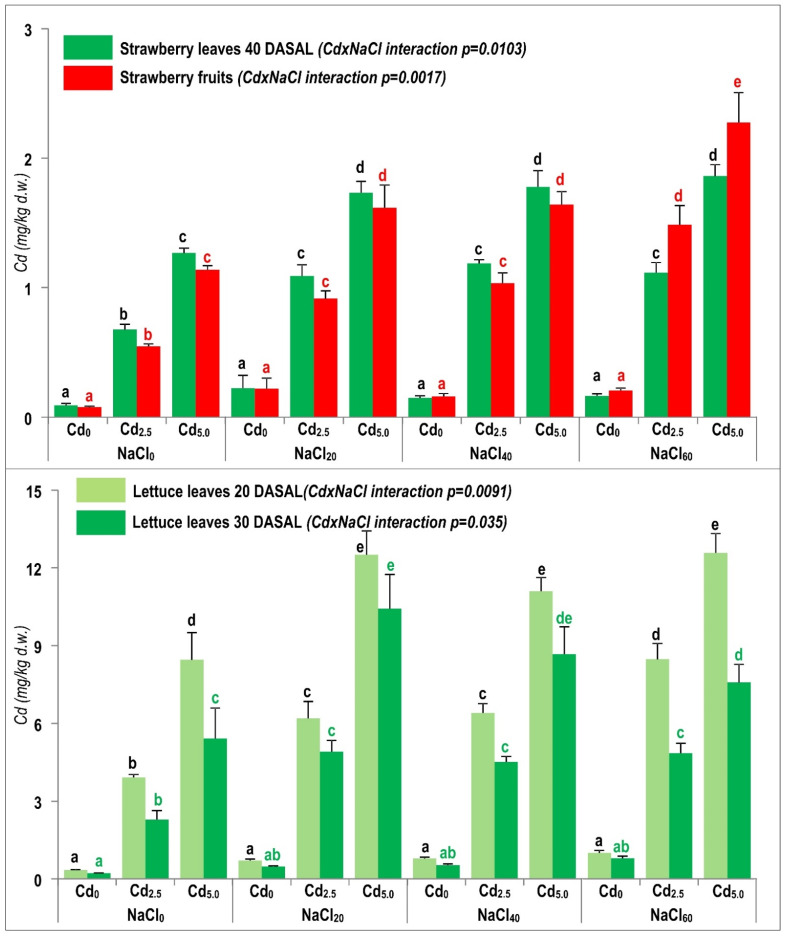
Impact of applied treatments on Cd concentration in lettuce leaves 20 and 30 days after salinity (DASAL) treatment commenced, and in strawberry leaves 40 DASAL and fruits. Bars (means + SE, *n* = 4) with the same letter and colour are not significantly different among the treatments according to the Tukey’s HSD test.

Figure 3 clearly shows strong dependence of Cd concentration in the crops and tissues on the applied combination of soil Cd contamination and NaCl salinity, i.e., the CdxNaCl interaction was significant (*p* = 0.0017–0.035). For instance, compared to the control (NaCl_0_ + Cd_0_) treatment, Cd concentration increased by 19-fold in strawberry leaves at 40 DASAL, and by 23-fold in marketable fruit tissue in the Cd_5.0_xNaCl_60_ treatment (Figure 3). Tissue Cd concentrations were higher in leaves than fruits in the treatments with up to 40 mM NaCl, whereas the opposite was true in the treatments with 60 mM NaCl (Figure 3). In lettuce leaves in general, Cd concentrations were higher (by 17–43%) at 20 DASAL in comparison with 30 DASAL in all treatments, and were 42-fold (20 DASAL) and 38-fold (30 DASAL) higher in Cd_5.0_xNaCl_60_ in comparison with the control (Figure 3). Additionally, at both sampling times, Cd leaf concentrations were markedly higher in lettuce than strawberry leaves (by 5.4- to 6.6-fold) or strawberry fruits (by 4.5- to 5.4-fold). NaCl salinity slightly (non-significantly) enhanced Cd accumulation in above-ground tissues in the non-contaminated (background) control (Cd_0_) treatment (Figure 3).

#### 2.4.1. Interaction of NaCl and Cd Stresses as Influenced by the Specific Properties of Tested Crops

Across all the treatments, Cd concentrations in strawberry leaves (0.08–2.3 mg/kg) were substantially lower than in lettuce leaves (0.22–12.6 mg/kg) (Figure 3), but detrimental visible symptoms, followed by plant mortality, were more pronounced in strawberry (Figure 1). Quite similar Cd concentrations in strawberry fruits (up to 2.3 mg/kg) and leaves (up to 1.9 mg/kg) suggested that Cd mobility was similar in the xylem and the phloem, especially up to 40 mM NaCl; however, under severe NaCl stress (60 mM), Cd concentrations in strawberry fruits exceeded those in leaves (Figure 3). Thus, up to moderate salinity level (≤40 mM NaCl), strawberry appeared able to suppress Cd translocation from leaves to fruits. Compared to strawbery, lettuce had a much stronger capacity for Cd accumulation in the leaf tissues. In addition, strawberry appeared to have a good capacity to deposit Cd in leaves, where vacuolar compartmentation could be a mechanism to lower cytosolic Cd activity [52] and protect reproductive tissue (fruits) from Cd toxicity. Additionally, it appeared that, at up to 40 mM NaCl, strawberry was able to control the Cd leaf-to-fruit translocation, but such control collapsed due to a cumulative impact of both stresses.

Critical toxic Cd concentration in tissues of non-metalophytes (e.g., our test crops) was suggested to be about 10 mg Cd/kg d.w. [53]. Consequently, lettuce was likely exposed to combined NaCl and Cd toxicities (Table 2, Figure 3). However, in strawberry, which was compromised more by salinity than lettuce, irrespective of relatively low Cd concentrations (i.e., by about 4-fold lower than the putative toxic level), the effect of the additional Cd stress should not be excluded. In addition, which stress (salinity or Cd) (i) dominated, (ii) started earlier (iii) and/or was more detrimental to test crops, remains to be elucidated in future studies. In support of this suggestion, [22] reported that the interactive effects of NaCl and Cd were more pronounced when compared with their separate effects, even though both NaCl and Cd exposure generated oxidative stress in roots and leaves of pigeonpea as indicated by electrolyte leakage and membrane peroxidation, as well-reduced leaf hydration and a decreased content of major photosynthetic pigments (chlorophyll a and b, and their ratio).

Plants react similarly to NaCl and metal (Cd) stresses regarding growth and dry matter reduction, chlorosis, apical tip browning, increased electrolyte leakage, and production of ROS and/or osmolites [2,22,28,29]. For instance, in two sorghum cultivars exposed to Cd (0.5–11 mg/L) for 55 days, [28] noticed reduced plant height and leaf number, accompanied by increased leaf (up to ~7.0 mg/kg) and stem (up to ~11.0 mg/kg) Cd concentration, membrane damage (electrolytes leakage), massive production of H_2_O_2_, osmolites, and increased activities of antioxidative enzymes (SOD, POD, CAT) due to oxidative stress. Similar findings were reported under Cd stress (increased H_2_O_2_/MDA concentrations, electrolyte leakage, and activities of SOD, POD, CAT, GPX and/or APX), followed by reduced grain yield of maize cultivars [30], and shoot yield of wheat genotypes [54].

Our results confirmed that the two salt-sensitive, non-methalophyte crops tested differed in Cd accumulation in leafy tissues, in either control or stressed conditions (Figure 3). This species-specific trait [55] may be valuable in pre-screening and plant selection for resistance to combined stresses [13]. Well-known genotypic variation in below- and aboveground tissue Cd concentration has been reported in numerous species, including tobacco [56], wheat [54], rice and soybean [55], pigeonpea [22], maize [30], sorghum [28], radish [14] as well as lettuce [57]. Using 16 lettuce varieties, [57] recorded leaf Cd concentrations of 0.52–1.2 mg Cd/kg in plants grown in the non-contaminated soil and 8.2–10.7 mg Cd/kg in the Cd-treated plants, which are similar concentrations to those reported in the present study (Figure 3). The same authors concluded that lettuce accumulated Cd predominantly in leaves (similarly to tobacco), with the uptake of K, Ca, Mg, N and P, as well as fresh and dry yield of shoots and roots not affected by relatively high Cd (0.1 mg/L) concentration in the rhizosphere solution. Recently, [2] reported that semi-halophyte Mesembryanthemum crystallinum stressed by NaCl (0.4 M) and Cd (10 mM) accumulated more Cd in comparison to plants not exposed to NaCl, likely due to the NaCl- and Cd-enhanced expression of genes (irt2, cax4, hma4, pcs1 and zip4) pivotal for metal uptake and (re)translocation in stressed plants. In particular, the irt2 expression was impacted by NaCl and Cd stresses cumulatively. In the future studies, it would be interesting to explore the individual and combined effects of NaCl and Cd at various rates on the expression of genes associated with Cd uptake and transport in lettuce and strawberry.

#### 2.4.2. Biogeochemistry of NaCl and Cd in Organically Rich Soil

In both trials, we used organically rich (>900 g/kg OM) peat soil (Table 3), widely used in fruit and vegetable production, in controlled environments [58,59], including in Croatia where almost complete greenhouse strawberry production is based on similar peat-based media. Such media are rich in complex humic substances (fulvates, humates), comprising many functional groups (carboxylic, phenolic and hydroxyl), underpinning high-capacity sorption (e.g., CEC >90 cmol/kg) of cationic metal forms [15,16,59] and formation of metallo-organic complexes [60] by humic substances. For instance, peat addition to mineral Cambisols can increase their OM content (by >6-fold) and CEC (by >3-fold), markedly increasing the adsorption of Cd (from 261 to 631 μg/g) and Pb (from 624 to 1270 μg/g) [15]; similarly, the addition of 1 g peat to aqueous solution removed 40 cmol/kg Cd [16]. Moreover, peats are very effective in metal immobilisation in highly contaminated (mine site) soils. For instance, adding peat to highly Pb-contaminated soil (2 g Pb/kg) reduced Pb leaching (up to >95%), mobility (up to 58%) and toxicity (up to >96%) [61], whereas the addition of peat to mining-contaminated soil (total Cd 2.7 mg/kg) increased soil Cd immobilisation (by 29–44%) and decreased its bioaccumulation in spinach (by 23–58%) in comparison to non-amended soil [5]. In contrast, in our trials, the expected ameliorative effects of OM-rich peat soil at relatively similar level of Cd contamination (total Cd up to 5.0 mg/kg) did not eventuate (Figure 3). It is likely that the interactions with NaCl resulted in the biogeochemistry of Cd, as well Cu and Zn (Table 2), that improved the uptake of these three elements by both test species.

The current understanding of Cd transfer from soil into roots and transport to shoots is still relatively poor and is based on numerous assumptions and postulates applicable to essential elements that are physicochemically similar to non-essential and toxic Cd (e.g., Zn and Cu, discussed below). Soluble Cd forms, such as free cationic Cd^2+^ and Cd–Cl complexes (CdCls), are presumed to be the most phytoavailable and, thus, toxic (Lund et al., 1990), and they dominate the acidic rhizosphere [12]. In the NaCl-loaded rhizospheres, the concentration of dissolved Cl was sufficient (>10 mM) to form relatively stable and mobile CdCl complexes [62]. In the study presented here, the formation of CdCl complexes might have been enhanced by increasing rates of NaCl and Cd applied (Table 3), which likely increased Cd uptake by the tested crops (Figure 3); this uptake could have been of CdCls directly and/or of free Cd^2+^ dissociated from CdCl complexes that had been transported to the root surface [14,63]. Recently, [13] reported that NaCl salinity affected Cd speciation in OM-enriched soil solution by promoting the formation of CdCl complexes, as well as by the significant NaClxCd interaction influencing Cd availability (CaCl_2_-extractable Cd) in the rhizosphere; however, Cd uptake and accumulation in faba bean were decreased.

Another important variable that plausibly played an important role in driving Cd (also Zn and Cu) mobility and phytoavailability was pH in the rhizosphere of strawberry and lettuce. In this study, pH in pots fluctuated in a narrow and slightly acidic range (5.80–6.15 in case of strawberry and 5.65–6.10 in case of lettuce; data not shown), contrasting with the study by [13] where pH was slightly alkaline (7.56–7.72), which might additionally have contributed in the latter study to soil immobilisation of Cd and the suppressed soil-faba bean transfer. Even under slightly alkaline conditions, metal immobilisation can be expected, due to precipitation as Cd(OH)_2_, Zn(OH)_2_ [6] or other inorganic metal salts, such as carbonates, sulfates and phosphates [12]. Given the high natural abundance of mostly carboxylic functional groups in the humic matrix (explained above), peats and their water solutions are acidic (pH < 6.0), thus facilitating the prevalence of mobile metallic species (Cd^2+^, Zn^2+^, Cu^2+^) [6,60]. Ref. [59] also noticed pronounced mobility and bioavailability of Cd, Cu and Pb in metal-contaminated soil associated with soil acidification due to OM (peat moss) addition. Accordingly, in the present study the acidic rhizosphere favoured the mobilisation of Cd, Cu and Zn, and with other pedovariables (e.g., increased Cl^−^) and enhanced metal uptake by strawberry and lettuce.

Uptake and root-shoot Cd transport can be affected by the synergistic or antagonistic interactions with similar nutrients, such as Zn, Cu or Mn [55]. As they are readily water-soluble and bioavailable, non-essential Cd ions may enter the root cells via nutrient (e.g., Zn or Fe) transporters in the plasma membrane (e.g., ZIP, IRT) [2,12,64]. In the present study, the applied NaCl and Cd treatments affected the biogeochemistry of Zn and Cu and their uptake and phytoaccumulation in the examined tissues (Table 2). Similarly, [55] suggested that the physiological mechanisms governing the uptake of Cd and its translocation to shoots of 11 rice cultivars could be associated with the uptake of essential metals that are chemically related. In the salt-sensitive (but not salt-tolerant) wheat genotype, the combined NaCl and Cd stresses led to increased Zn concentration in leaves, whereas higher Cu (and Mn) leaf concentrations were found only in the salt-tolerant genotype [54]. Under non-stress conditions, it would be expected that, due to competition effects, increased concentration of one trace metal, i.e., Cd, might suppress the rhizosphere-to-plant transfer of chemically similar micronutrient metals (e.g., Zn and/or Cu), as confirmed in the present study for Cu (tissue concentration decreased by up to 17% in strawberry and by up to 20% in lettuce) and Zn (tissue concentration decreased by up to 16% in strawberry and by up to 18% in lettuce) under Cd exposure (Table 2). However, in the present study, under NaCl stress conditions, whereby even the uptake and accumulation of macronutrients were compromised (Table 2, Figure 2), disturbed mineral relations (e.g., soil saturation with Na^+^) might have additionally favoured micronutrient Zn^2+^/Cu^2+^ desorption in the rhizosphere soil and their increased uptake, e.g., Zn up to ~70% in strawberry and up to >150% in lettuce, and Cu up to 90% in strawberry and up to 120% in lettuce (Table 2). Finally, (non)specific macro/micronutrient membrane transporters/channels could also have contributed to the uptake of Zn^2+^, Cu^2+^, Cd^2+^ and/or CdCls under NaCl+Cd exposure in the present study (Table 2, Figure 3), but further work is needed.

## 3. Materials and Methods

### 3.1. Experimental Setup and Conditions

Two controlled experiments, with lettuce and strawberry, were performed in a polyethylene greenhouse at the experimental station of Faculty of Agriculture, University of Zagreb, Croatia (16°02′ E; 45°50′ N, 128 m.a.s.l.). A peat growth medium (Potground H, Klasmann-Deilmann GmbH, Geeste, Germany) was air-dried and sieved through a 2-mm mash, and was then spiked with a stock solution of Cd(NO_3_)_2_ × 4 H_2_O at two rates (to achieve the total Cd soil concentrations of 2.5 and 5.0 mg Cd/kg d.w.) in addition to the control with a background concentration of 0.33 (±0.01) mg Cd/kg d.w. (Table 3) according to the procedure explained in detail elsewhere [13,21]. The prepared substrate was thoroughly mixed once a week to improve homogeneity. After 5 weeks of substrate incubation, uniform 2-week-old lettuce (*Lactuca sativa* L., cv Tiffany) and frigo A+ strawberry (*Fragaria ananassa* Duch., cv Elsanta) seedlings were transplanted into the 2 L plastic pots (one seedling/pot) containing incubated peat growth medium.

During the first week after transplanting, in the case of lettuce, and the first two weeks in the case of strawberry, seedlings were fertigated with a standard nutrient solution (Table 3). Thereafter, salinity treatment was imposed by dissolving NaCl in the standard nutrient solution at four rates: 20, 40 and 60 mM, plus control without NaCl (Table 3), with a gradual (10 mM daily) increase to full concentration. Fertigation management (rate, frequency) was identical for all the treatments using drip irrigation (CNL 2 L/h, Netafim Ltd., Kibutz Hatzerim, Israel) and maintaining electrical conductivity (EC) at 2.0 (±0.1) dS/m in the rhizosphere (at 15 cm depth) of the control treatment (measurements by an EC-soil probe (EcoScan CON 5)).

### 3.2. Data Collection, Sampling and Chemical Analyses

During the experiments, lettuce leaf samples were collected 20 and 30 days after salinity (DASAL) treatment commenced, whereas strawberry leaves were collected 40 DASAL, and fruits were collected (from the beginning of ripening to the end of experiment) as technologically ripe (market ready). Strawberry runners were not pruned, but were counted, and the length of the longest runner was measured at the end of the trial. Each leaf sample contained two fully developed mature leaves from each plant. Strawberry leaves were scanned (Umax, PowerLook III, Taiwan) and processed in ImageJ 1.52q v. software to calculate leaf area. Thereafter, plant materials were weighed fresh and after drying (48 h at 65 °C), and were powdered in an inox grinder (Krups, Bochum, Germany) prior to chemical analyses. Approximately 0.2 g of powdered plant material was digested in a mixture of HNO_3_ and H_2_O_2_ (*v*/*v* = 5:1), and total concentrations of Ca, Mg, Cd, Cu and Zn were detected by inductively coupled plasma–atomic emission spectrometry (Vista, MPX AX, Varian, Melbourne, Victoria, Australia). In the deionised water extract of various plant tissues, chlorides were detected by a colourimetric assay at 470 nm in an automated continuous flow autoanalyser (San++, Skalar Analytical, Breda, Netherlands). The K/Na, Ca/Na and Mg/Na ratios in plant tissues were calculated based on the (milli) equivalent dry weight basis. The analytical quality control procedure comprised reagent blanks, duplicate samples and the reference plant materials from the inter-laboratory calibration program [13].

### 3.3. Experimental Design and Statistical Analysis

Both experiments were set up in a completely randomised block design with 12 combined treatments (4 NaCl × 3 Cd) (Table 3) in four replications. The significance (*p* value) of the main effects and their interaction (CdxNaCl) for all measured growth and mineral accumulation parameters in both crops was tested by the two-way analysis of variance (ANOVA) using the statistical software package SAS ver. 9.1.3 [65]. The significance of differences among the means of applied treatments was determined using the Tukey’s HSD test.

## 4. Conclusions

The combined NaCl-Cd exposure had detrimental effects on (i) vegetative (e.g., reduced commercial yield, leaf area, number and length of runners), (ii) physiological (e.g., leaf tip burns and chlorosis/necrosis, shortened vegetation, plant death), and (iii) mineral uptake parameters (e.g., over-accumulation of Na^+^ and Cl^−^ followed by a sharp decline in K, Ca and Mg concentrations in examined tissues) using widely consumed glycophytic fruit (strawberry) and leafy (lettuce) horticultural crops. The NaCl treatment markedly enhanced the accumulation of micronutrients Zn and Cu, and especially of toxic Cd in edible tissues. The Cd concentration in lettuce exceeded phytotoxic level (>12 mg/kg), suggesting a high capacity to take up Cd in a short period as well as a poor capacity to prevent Cd translocation to edible parts. In contrast, compared with lettuce, Cd concentration in strawberry was several-fold lower, but similar to strawberry fruits and leaves, suggesting its higher potential (vs. lettuce) to control Cu uptake and root-to-shoot transfer. However, at ≥40 mM NaCl exposure, strawberry could not restrict Cd transport via phloem from leaves to fruits. Such outcomes might have been due to the biogeochemical reactions in the Na^+^-saturated humics-rich and acidic rhizosphere, whereby the pronounced complexation of Cd with Cl^−^ and organic ligands facilitated metal mobility and phytoavailability by minimizing Cd, Cu and Zn soil sorption.

## Figures and Tables

**Table 1:** Impact of applied treatments on vegetative growth and yield parameters of test plant species 70 days (strawberry) and 30 days after salinity treatment commenced (lettuce). Means (standard errors in parenthesis; *n* = 4) with the same letter in a column for a given main effect are not significantly different, according to the Tukey’s HSD test.

Treatment	Strawberry					Lettuce
	Marketable Fruit Number/Plant	Total Marketable Fruit g/Plant	Total Runners Number/Plant	The Longest Runner cm	Leaf Area cm^2^	Head Weight g/Plant
Cd_0_	7.8(0.67) a	62(6.5) a	2.6(0.37) a	54(5.3) a	171(13) a	399(13) a
Cd_2.5_	7.3(0.56) a	63(6.3) a	2.0(0.31) ab	48(3.8) ab	174(13) a	376(14) ab
Cd_5.0_	8.3(0.58) a	74(7.3) a	1.9(0.28) b	46(4.6) b	164(10) a	359(11) b
NaCl_0_	10(0.54) a	96(6.0) a	3.5(0.29) a	71(2.6) a	235(8.0) a	422(10) a
NaCl_20_	7.8(0.57) b	69(5.8) b	2.7(0.21) b	54(3.8) b	180(8.0) b	411(7.0) a
NaCl_40_	7.5(0.49) bc	63(4.8) b	1.9(0.21) b	44(1.9) c	154(5.4) c	367(10) b
NaCl_60_	5.7(0.44) c	39(3.9) c	0.5(0.08) c	27(2.4) d	111(3.0) d	311(8.0) c
NaClxCd	ns	ns	ns	ns	ns	ns

**Table 2:** Impact of NaCl and Cd treatments on dry matter (DM) percentage and element concentrations in strawberry and lettuce tissues 20, 30 and 40 days after salinity (DASAL) treatment commenced. Means (standard errors in parenthesis; *n* = 4) with the same letter in a column for a given main effect are not significantly different according to the Tukey’s HSD test.

Treatment	Strawberry Leaves 40 DASAL					Lettuce Leaves 20 DASAL				
	DM %	Na	Cl	Cu	Zn	DM %	Na	Cl	Cu	Zn
		g/kg		mg/kg			g/kg		mg/kg	
Cd_0_	23(1.0) a	5.0(1.2) a	26(3.75) a	4.7(0.28) a	33(2.0) a	4.9(0.07) a	21(3.2) a	47(6.9) a	4.9(0.30) a	77(3.7) a
Cd_2.5_	22(0.73) b	4.0(0.87) b	23(3.40) b	4.4(0.28) a	31(1.7) ab	5.0(0.07) a	21(3.3) a	48(7.0) a	4.8(0.31) ab	78(4.3) a
Cd_5.0_	22(0.77) b	4.0(0.89) b	22(3.20) b	3.9(0.20) a	28(0.90) b	5.0(0.08) a	20(3.1) a	47(6.5) a	4.4(0.33) b	71(4.7) b
NaCl_0_	19(0.32) a	0.10(0.001) a	2.9(0.30) a	4.4(0.36) a	23(0.91) a	4.9(0.10) ab	1.9(0.06) a	6.4(0.23) a	3.2(0.13) a	53(1.7) a
NaCl_20_	21(0.33) ab	2.4(0.14) b	23(0.82) b	4.2(0.32) a	32(1.1) b	4.7(0.10) b	18(0.27) b	46(0.89) b	4.0(0.11) b	71(1.8) b
NaCl_40_	22(0.47) b	4.7(0.30) c	30(1.10) c	4.3(0.28) a	34(1.5) b	4.9(0.05) ab	27(0.40) c	61(2.4) c	5.5(0.22) c	83(1.4) c
NaCl_60_	27(0.77) c	10(0.46) d	39(1.05) d	4.6(0.27) a	34(2.1) b	5.1(0.06) b	35(0.70) d	76(1.9) d	6.0(1.14) d	95(2.4) d
CdxNaCl	ns	*	ns	ns	ns	ns	ns	ns	ns	ns
**Treatment**	**Strawberry Fruits**					**Lettuce Leaves 30 DASAL**				
	**DM** **%**	**Na**	**Cl**	**Cu**	**Zn**	**DM** **%**	**Na**	**Cl**	**Cu**	**Zn**
		**g/kg**		**mg/kg**			**g/kg**		**mg/kg**	
Cd_0_	7.9(0.20) a	3.3(0.81) a	13(2.2) a	4.5(0.33) a	24(1.4) a	5.8(0.14) a	34(5.5) a	63(9.7) a	6.2(0.46) a	107(9.6) a
Cd_2.5_	8.1(0.14) a	3.0(0.60) a	11(1.5) ab	3.9(0.25) b	20(1.0) b	5.6(0.13) a	35(5.3) a	62(9.4) a	5.8(0.47) a	96(7.0) ab
Cd_5.0_	8.2(0.22) a	2.8(0.75) a	10(1.7) b	4.0(0.32) b	20(1.0) b	5.6(0.11) a	33(5.0) a	59(8.7) a	4.9(0.46) b	88(8.2) b
NaCl_0_	8.2(0.07) ab	0.2(0.01) a	3.2(0.30) a	3.1(0.13) a	16(0.77) a	5.9(0.15) a	2.0(0.03) a	6.5(0.15) a	3.5(0.20) a	54(3.0) a
NaCl_20_	8.3(0.27) a	2.0(0.30) b	7.8(0.58) b	3.5(0.15) ab	21(1.0) b	5.3(0.12) b	33(0.80) b	58(1.3) b	4.6(0.20) b	93(3.0) b
NaCl_40_	8.1(0.22) ab	3.0(0.27) b	12(0.92) c	4.1(0.13) b	22(1.0) b	5.5(0.17) ab	45(1.7) c	79(2.9) c	6.7(0.36) c	105(2.5) b
NaCl_60_	7.6(0.19) b	7.1(0.64) c	21(1.3) d	5.9(0.26) c	27(1.0) c	5.9(0.08) a	56(1.8) d	102(3.3) d	7.7(0.30) d	136(8.2) c
CdxNaCl	ns	ns	ns	ns	ns	ns	ns	ns	ns	ns

* at *p* < 0.05; ns, not significant.

**Table 3:** Chemical composition of standard nutrient solution, peat soil and the applied treatments. The values in parentheses represent standard errors (*n* = 3).

Nutrient Solution	Concentration	Peat Soil
pH	6.5(±0.1)	5.23 (±0.2)
OM		902 (±61) g/kg
EC	1.7(±0.1) dS/m	
NO_3_^−^	10 mM	
H_2_PO_4_^−^	1.5 mM	
K^+^	7.5 mM	
Ca^2+^	2.0 mM	
Mg^2+^	2.0 mM	
SO_4_^2−^	2.0 mM	
Fe	15 µM	
Mn	10 µM	
Total Cu	10 µM	12.9 (±0.06) mg/kg
B	5 µM	
Total Zn	0.7 µM	14.7 (±0.04) mg/kg
Mo	0.7 µM	
Total Cd		0.33 (±0.01) mg/kg
Salinity treatment	0 (NaCl_0_), 20 (NaCl_20_), 40 (NaCl_40_) and 60 (NaCl_60_) mM NaCl	
Cd treatment	0.33 (±0.01; Cd_0_), 2.5 (±0.15; Cd_2.5_) and 5.0 (±0.20; Cd_5.0_) mg/kg	

## Data Availability

Not applicable.

## Supplementary Materials

The following are available online at https://www.mdpi.com/article/10.3390/plants10061202/s1.

## References

[1] Ondrasek G., Bakić Begić H., Romić D., Brkić Ž., Husnjak S., Bubalo Kovačić M. (2021). A novel LUMNAqSoP approach for prioritising groundwater monitoring stations for implementation of the Nitrates Directive. Environ. Sci. Eur..

[2] Nosek M., Kaczmarczyk A., Jędrzejczyk R.J., Supel P., Kaszycki P., Miszalski Z. (2020). Expression of Genes Involved in Heavy Metal Trafficking in Plants Exposed to Salinity Stress and Elevated Cd Concentrations. Plants.

[3] Ondrasek G. (2014). Water Scarcity and Water Stress in Agriculture. Physiological Mechanisms and Adaptation Strategies in Plants Under Changing Environment.

[4] Ondrasek G., Rengel Z. (2021). Environmental salinization processes: Detection, implications & solutions. Sci. Total Environ..

[5] Nawab J., Khan N., Ahmed R., Khan S., Ghani J., Rahman Z., Khan F., Wang X., Muhammad J., Sher H. (2019). Influence of different organic geo-sorbents on Spinacia oleracea grown in chromite mine-degraded soil: A greenhouse study. J. Soils Sediments.

[6] Egene C.E., Van Poucke R., Ok Y.S., Meers E., Tack F.M.G. (2018). Impact of organic amendments (biochar, compost and peat) on Cd and Zn mobility and solubility in contaminated soil of the Campine region after three years. Sci. Total Environ..

[7] Dell’Aversana E., Hessini K., Ferchichi S., Fusco G.M., Woodrow P., Ciarmiello L.F., Abdelly C., Carillo P. (2021). Salinity Duration Differently Modulates Physiological Parameters and Metabolites Profile in Roots of Two Contrasting Barley Genotypes. Plants.

[8] Sofo A., Scopa A., Nuzzaci M., Vitti A. (2015). Ascorbate Peroxidase and Catalase Activities and Their Genetic Regulation in Plants Subjected to Drought and Salinity Stresses. Int. J. Mol. Sci..

[9] Munns R., Tester M. (2008). Mechanisms of Salinity Tolerance. Annu. Rev. Plant Biol..

[10] Mulaudzi T., Hendricks K., Mabiya T., Muthevhuli M., Ajayi R.F., Mayedwa N., Gehring C., Iwuoha E. (2020). Calcium Improves Germination and Growth of Sorghum bicolor Seedlings under Salt Stress. Plants.

[11] Ferreira J.F.S., da Silva Filho J.B., Liu X., Sandhu D. (2020). Spinach Plants Favor the Absorption of K+ over Na+ Regardless of Salinity, and May Benefit from Na+ When K+ is Deficient in the Soil. Plants.

[12] Ondrasek G. (2013). The responses of salt-affected plants to cadmium. Salt Stress in Plants: Signalling, Omics and Adaptations.

[13] Filipović L., Romić M., Romić D., Filipović V., Ondrašek G. (2018). Organic matter and salinity modify cadmium soil (phyto)availability. Ecotoxicol. Environ. Saf..

[14] Ondrasek G., Romic D., Rengel Z. (2020). Interactions of humates and chlorides with cadmium drive soil cadmium chemistry and uptake by radish cultivars. Sci. Total Environ..

[15] Pelinsom Marques J., Silvestre Rodrigues V.G., Monici Raimondi I., Zanin Lima J. (2020). Increase in Pb and Cd Adsorption by the Application of Peat in a Tropical Soil. Water Air Soil Pollut..

[16] Fine P., Scagnossi A., Chen Y., Mingelgrin U. (2005). Practical and mechanistic aspects of the removal of cadmium from aqueous systems using peat. Environ. Pollut..

[17] Bartczak P., Norman M., Klapiszewski Ł., Karwańska N., Kawalec M., Baczyńska M., Wysokowski M., Zdarta J., Ciesielczyk F., Jesionowski T. (2018). Removal of nickel(II) and lead(II) ions from aqueous solution using peat as a low-cost adsorbent: A kinetic and equilibrium study. Arab. J. Chem..

[18] Ferreira J.F.S., Liu X., Suarez D.L. (2019). Fruit yield and survival of five commercial strawberry cultivars under field cultivation and salinity stress. Sci. Hortic..

[19] Chinnusamy V., Jagendorf A., Zhu J.-K. (2005). Understanding and Improving Salt Tolerance in Plants. Crop Sci..

[20] Ul Haq T., Akhtar J., Steele K.A., Munns R., Gorham J. (2014). Reliability of ion accumulation and growth components for selecting salt tolerant lines in large populations of rice. Funct. Plant Biol..

[21] Ondrasek G., Davor R., Zed R., Marija R., Monika Z. (2009). Cadmium accumulation by muskmelon under salt stress in contaminated organic soil. Sci. Total Environ..

[22] Garg N., Chandel S. (2012). Role of Arbuscular Mycorrhizal (AM) Fungi on Growth, Cadmium Uptake, Osmolyte, and Phytochelatin Synthesis in Cajanus cajan (L.) Millsp. Under NaCl and Cd Stresses. J. Plant Growth Regul..

[23] Akrami M., Arzani A. (2019). Inheritance of fruit yield and quality in melon (Cucumis melo L.) grown under field salinity stress. Sci. Rep..

[24] Kaya C., Higgs D., Saltali K., Gezerel O. (2002). Response of Strawberry Grown at High Salinity and Alkalinity to Supplementary Potassium. J. Plant Nutr..

[25] Bose J., Munns R., Shabala S., Gilliham M., Pogson B., Tyerman S.D. (2017). Chloroplast function and ion regulation in plants growing on saline soils: Lessons from halophytes. J. Exp. Bot..

[26] Tester M., Davenport R. (2003). Na+ Tolerance and Na+ Transport in Higher Plants. Ann. Bot..

[27] Farooq M.A., Niazi A.K., Akhtar J., Saifullah, Farooq M., Souri Z., Karimi N., Rengel Z. (2019). Acquiring control: The evolution of ROS-Induced oxidative stress and redox signaling pathways in plant stress responses. Plant Physiol. Biochem..

[28] Jawad Hassan M., Ali Raza M., Ur Rehman S., Ansar M., Gitari H., Khan I., Wajid M., Ahmed M., Abbas Shah G., Peng Y. (2020). Effect of Cadmium Toxicity on Growth, Oxidative Damage, Antioxidant Defense System and Cadmium Accumulation in Two Sorghum Cultivars. Plants.

[29] Kim Y.-H., Khan A.L., Kim D.-H., Lee S.-Y., Kim K.-M., Waqas M., Jung H.-Y., Shin J.-H., Kim J.-G., Lee I.-J. (2014). Silicon mitigates heavy metal stress by regulating P-type heavy metal ATPases, Oryza sativalow silicon genes, and endogenous phytohormones. BMC Plant Biol..

[30] Anjum S.A., Tanveer M., Hussain S., Bao M., Wang L., Khan I., Ullah E., Tung S.A., Samad R.A., Shahzad B. (2015). Cadmium toxicity in Maize (*Zea mays* L.): Consequences on antioxidative systems, reactive oxygen species and cadmium accumulation. Environ. Sci. Pollut. Res..

[31] Pang Q., Chen X., Lv J., Li T., Fang J., Jia H. (2020). Triacontanol Promotes the Fruit Development and Retards Fruit Senescence in Strawberry: A Transcriptome Analysis. Plants.

[32] Marschner H. (2011). Marschner’s Mineral Nutrition of Higher Plants.

[33] Furumoto T., Yamaguchi T., Ohshima-Ichie Y., Nakamura M., Tsuchida-Iwata Y., Shimamura M., Ohnishi J., Hata S., Gowik U., Westhoff P. (2011). A plastidial sodium-dependent pyruvate transporter. Nature.

[34] Geilfus C.-M. (2018). Chloride: From Nutrient to Toxicant. Plant Cell Physiol..

[35] White P.J., Broadley M.R. (2001). Chloride in soils and its uptake and movement within the plant: A review. Ann. Bot..

[36] Xu G., Magen H., Tarchitzky J., Kafkafi U., Sparks D.L. (1999). Advances in Chloride Nutrition of Plants. Advances in Agronomy.

[37] Sun Y., Niu G., Wallace R., Masabni J., Gu M. (2015). Relative Salt Tolerance of Seven Strawberry Cultivars. Horticulturae.

[38] Grattan S., Grieve C. (1998). Salinity–mineral nutrient relations in horticultural crops. Sci. Hortic..

[39] Al-Khateeb S.A. (2006). Effect of Calcium/Sodium Ratio on Growth and Ion Relations of Alfalfa (Medicago sativa L.) Seedling Grown under Saline Condition. J. Agron..

[40] Fuad Mondal M., Asaduzzaman M., Ueno M., Kawaguchi M., Yano S., Ban T., Tanaka H., Asao T. (2017). Reduction of Potassium (K) Content in Strawberry Fruits through KNO3 Management of Hydroponics. Hortic. J..

[41] Assaha D.V.M., Ueda A., Saneoka H., Al-Yahyai R., Yaish M.W. (2017). The Role of Na+ and K+ Transporters in Salt Stress Adaptation in Glycophytes. Front. Physiol..

[42] Shi H., Lee B., Wu S.-J., Zhu J.-K. (2003). Overexpression of a plasma membrane Na+/H+ antiporter gene improves salt tolerance in Arabidopsis thaliana. Nat. Biotechnol..

[43] Horie T. (2004). Sodium Transporters in Plants. Diverse Genes and Physiological Functions. Plant Physiol..

[44] Hauser F., Horie T. (2010). A conserved primary salt tolerance mechanism mediated by HKT transporters: A mechanism for sodium exclusion and maintenance of high K +/Na + ratio in leaves during salinity stress. Plant. Cell Environ..

[45] Rengel Z. (1992). The role of calcium in salt toxicity. Plant Cell Environ..

[46] Pottosin I., Dobrovinskaya O. (2015). Ion Channels in Native Chloroplast Membranes: Challenges and Potential for Direct Patch-Clamp Studies. Front. Physiol..

[47] Khorshidi M.B., Yarnia M., Hassanpanah D. (2009). Salinity effect on nutrients accumulation in alfalfa shoots in hydroponic condition. J. Food Agric. Environ..

[48] Khayyat M., Rajaee S., Abdoreza S., Eshghi S., Tafazoli E. (2009). Calcium effects on changes in chlorophyll contents, dry weight and micronutrients of strawberry (Fragaria × ananassa Duch.) plants under salt-stress conditions. Fruits.

[49] Meriño-Gergichevich C., Ondrasek G., Zovko M., Šamec D., Alberdi M., Reyes-Díaz M. (2015). Comparative study of methodologies to determine the antioxidant capacity of Al-toxified blueberry amended with calcium sulfate. J. Soil Sci. Plant Nutr..

[50] Tahjib-Ul-Arif M., Roy P.R., Al Mamun Sohag A., Afrin S., Rady M.M., Hossain M.A. (2018). Exogenous Calcium Supplementation Improves Salinity Tolerance in BRRI Dhan28; a Salt-Susceptible High-Yielding Oryza Sativa Cultivar. J. Crop Sci. Biotechnol..

[51] Hayes P.E., Clode P.L., Guilherme Pereira C., Lambers H. (2019). Calcium modulates leaf cell-specific phosphorus allocation in Proteaceae from south-western Australia. J. Exp. Bot..

[52] Sharma S.S., Dietz K.-J., Mimura T. (2016). Vacuolar compartmentalization as indispensable component of heavy metal detoxification in plants. Plant. Cell Environ..

[53] Cabrera D., Young S.D., Rowell D.L. (1988). The toxicity of cadmium to barley plants as affected by complex formation with humic acid. Plant Soil.

[54] Mühling K.H., Läuchli A. (2003). Interaction of NaCl and Cd stress on compartmentation pattern of cations, antioxidant enzymes and proteins in leaves of two wheat genotypes differing in salt tolerance. Plant Soil.

[55] Ishikawa S., Ae N., Sugiyama M., Murakami M., Arao T. (2005). Genotypic Variation in Shoot Cadmium Concentration in Rice and Soybean in Soils with Different Levels of Cadmium Contamination. Soil Sci. Plant Nutr..

[56] Clarke B.B., Brennan E. (1989). Differential Cadmium Accumulation and Phytotoxicity in Sixteen Tobacco Cultivars. JAPCA.

[57] Florijn P.J., Nelemans J.A., Beusichem M.L. (1991). van Cadmium uptake by lettuce varieties. Neth. J. Agric. Sci..

[58] Cristiano G., Vuksani G., Tufarelli V., De Lucia B. (2018). Response of Weeping Lantana (Lantana montevidensis) to Compost-Based Growing Media and Electrical Conductivity Level in Soilless Culture: First Evidence. Plants.

[59] Park J.H., Lee S.-J., Lee M.-E., Chung J.W. (2016). Comparison of heavy metal immobilization in contaminated soils amended with peat moss and peat moss-derived biochar. Environ. Sci. Process. Impacts.

[60] Ondrasek G., Rengel Z. (2012). The Role of Soil Organic Matter in Trace Element Bioavailability and Toxicity. Abiotic Stress Responses in Plants.

[61] Lee S.-J., Lee M.-E., Chung J.W., Park J.H., Huh K.Y., Jun G.-I. (2013). Immobilization of Lead from Pb-Contaminated Soil Amended with Peat Moss. J. Chem..

[62] López-Chuken U.J., López-Domínguez U., Parra-Saldivar R., Moreno-Jiménez E., Hinojosa-Reyes L., Guzmán-Mar J.L., Olivares-Sáenz E. (2012). Implications of chloride-enhanced cadmium uptake in saline agriculture: Modeling cadmium uptake by maize and tobacco. Int. J. Environ. Sci. Technol..

[63] Smolders E. (1996). Effect of Cl on Cd uptake by Swiss chard in nutrient solutions. Plant Soil.

[64] Milner M.J., Seamon J., Craft E., Kochian L. (2013). V Transport properties of members of the ZIP family in plants and their role in Zn and Mn homeostasis. J. Exp. Bot..

[65] SAS Institution Inc. (2004). SAS v. 9.1.3.

