# Supplementary material for: Growth and Element Uptake by Salt-Sensitive Crops under Combined NaCl and Cd Stresses
Source: Plants (Basel). 2021 Jun 12;10(6):1202. doi: 10.3390/plants10061202 (PMC8231652; doi:10.3390/plants10061202)
Supplement: Supplementary file 1 [file plants-10-01202-s001.zip › plants-1228449-supplementary.pdf]

Growth and Element Uptake by Salt-Sensitive Crops under Combined NaCl and Cd Stresses

Gabrijel Ondrasek [1,*], Zed Rengel [2,3], Nada Maurović [1], Nada Kondres [1], Vilim Filipović [1], Radovan Savić [4], Boško Blagojević [4], Vjekoslav Tanaskovik [5], Cristian Meriño Gergichevich [6] and Davor Romić [1]

[1] Department of Soil Amelioration, University of Zagreb Faculty of Agriculture, 10000 Zagreb, Croatia; nmaurovic@agr.hr (N.M.); nkondres@agr.hr (N.K.); vfilipovic@agr.hr (V.F.); dromic@agr.hr (D.R.)
[2] School of Earth and Environment, University of Western Australia, Perth 6009, Australia; zed.rengel@uwa.edu.au
[3] Institute for Adriatic Crops and Karst Reclamation, 21000 Split, Croatia
[4] Department of Water Management, Faculty of Agriculture, University of Novi Sad, 21102 Novi Sad, Serbia; radovan.savic@polj.uns.ac.rs (R.S.); bosko.blagojevic@polj.uns.ac.rs (B.B.)
[5] Faculty of Agricultural Sciences and Food, University of Ss. Cyril and Methodius in Skopje, 1000 Skopje, North Macedonia; vjekoslavtanaskovic@yahoo.com
[6] Departamento de Producción Agropecuaria, Facultad de Ciencias Agropecuarias y Forestales, Universidad de La Frontera, Temuco 1145, Chile; cristian.merino@ufrontera.cl
* Correspondence: gondrasek@agr.hr

Table S1. Summary results of ANOVA for vegetative growth and yield parameters of test plant species. Strawberry was harvested 70 days and lettuce 30 days after salinity treatment commenced

Source	*DF*	Strawberry: Marketable fruit number/plant *F*	*p*	Strawberry: Total marketable fruit g/plant *F*	*p*	Strawberry: Total runners number/plant *F*	*p*	Strawberry: The longest runner cm *F*	*p*	Strawberry: Leaf area cm² *F*	*p*	Lettuce: Head weight g/plant *F*	*p*
Cd	2	1.08	ns	2.07	ns	5.26	*	3.78	*	0.93	ns	8.87	*
NaCl	3	13	***	20	***	41	***	51.2	***	59	***	44	***
CdxNaCl	6	0.72	ns	0.53	ns	0.97	ns	1.39	ns	0.44	ns	1.82	ns

DF, degrees of freedom; *** at p<0.001; * at p<0.05; ns, not significant

Table S2. Summary results of ANOVA for dry matter (DM) percentage and element concentrations in strawberry and lettuce tissues 20, 30 and 40 days after salinity (DASAL) treatment commenced

Source	*DF*	*F*	*p*	*F*	*p*	*F*	*p*	*F*	*p*	*F*	*p*	*F*	*p*	*F*	*p*	*F*	*p*	*F*	*p*	*F*	*p*	*F*	*p*	*F*	*p*
		DM		Na		Cl		Cd		Cu		Zn		DM		Na		Cl		Cd		Cu		Zn	
				**Strawberry leaves 40 DASAL**												**Lettuce leaves 20 DASAL**									
Cd	2	6.11	*	9.60	*	14	***	464	***	2.23	ns	4.38	*	0.44	ns	1.03	ns	0.24	ns	355	***	5.59	*	8.15	*
NaCl	3	56	***	433	***	501	***	20	***	0.26	ns	14	***	4.63	*	194	***	327	***	17	***	87	***	124	***
CdxNaCl	6	1.4	ns	4.4	*	1.7	ns	3.3	*	0.32	ns	2.2	ns	0.66	ns	0.79	ns	0.48	ns	3.4	*	1.5	ns	0.96	ns
				**Strawberry fruits**												**Lettuce leaves 30 DASAL**									
Cd	2	0.73	ns	0.60	ns	4.01	*	203	***	5.43	*	7.15	*	0.93	ns	0.39	ns	1.13	ns	130	***	13.2	***	6.82	*
NaCl	3	3.1	*	53	***	93	***	24	***	60	***	26	***	5.18	*	281	***	296	***	8.6	*	83	***	65	***
CdxNaCl	6	0.22	ns	0.35	ns	1.5	ns	4.5	*	1.7	ns	1.9	ns	0.39	ns	0.53	ns	0.67	ns	2.6	*	1.60	ns	1.44	ns

DF, degrees of freedom; *** p<0.001; * p<0.05; ns, not significant

Table S3. Summary results of ANOVA for K/Na, Ca/Na and Mg/Na concentration ratios in lettuce leaves 20 and 30 days after salinity (DASAL) treatment commenced, and in strawberry leaves 40 DASAL and fruits.

Source	*DF*	*F*	*p*	*F*	*p*	*F*	*p*	*F*	*p*	*F*	*p*	*F*	*p*
		K/Na		Ca/Na		Mg/Na		K/Na		Ca/Na		Mg/Na	
		**Strawberry leaves 40 DASAL**						**Lettuce leaves 20 DASAL**					
Cd	2	0.59	ns	0.16	ns	0.82	ns	1.0	ns	1.6	ns	2.1	ns
NaCl	3	166	***	86	***	163	ns	749	***	681	***	138	***
CdxNaCl	6	0.67	ns	0.14	ns	0.81	ns	1.2	ns	1.2	ns	1.8	ns
		**Strawberry fruits**						**Lettuce leaves 30 DASAL**					
Cd	2	2.0	ns	3.0	ns	1.72	ns	0.15	ns	1.9	ns	0.7	ns
NaCl	3	321	**	295	**	313	**	502	***	540	***	180	***
CdxNaCl	6	1.8	ns	2.2	ns	1.5	ns	0.17	ns	1.9	ns	0.8	ns

DF, degrees of freedom; *** p<0.001; * p<0.05; ns, not significant
